# Application of stem cell-derived exosomes in ischemic diseases: opportunity and limitations

**DOI:** 10.1186/s12967-021-02863-w

**Published:** 2021-05-08

**Authors:** Majid Babaei, Jafar Rezaie

**Affiliations:** 1 Social Determinants of Health Research Center, Clinical Research Institute, Urmia University of Medical Sciences, Urmia, Iran; 2 Solid Tumor Research Center, Cellular and Molecular Medicine Research Institute, Urmia University of Medical Sciences, P.O. Box: 1138, 57147 Urmia, Iran

**Keywords:** Exosomes, Ischemic diseases, Angiogenesis, Extracellular vesicles

## Abstract

Ischemic diseases characterized by an insufficient blood flow that leads to a decrease in oxygen and nutrient uptake by cells have emerged as an important contributor to both disability and death worldwide. Up-regulation of angiogenesis may be a key factor for the improvement of ischemic diseases. This article searched articles in PubMed with the following keywords: stem cells, exosomes, angiogenesis, ischemic diseases either alone or in grouping form. The most relevant selected items were stem cell-derived exosomes and ischemic diseases. A growing body of evidence indicates that stem cells produce exosomes, which is the novel emerging approach to cell-to-cell communication and offers a new standpoint on known therapeutic strategies of ischemic diseases. Exosomes transport biological molecules such as many types of proteins, RNAs, DNA fragments, signaling molecules, and lipids between cells. Different stem cells release exosomes representing beneficial effects on ischemic diseases as they promote angiogenesis both in vitro and in vivo experiments. Application of exosomes for therapeutic angiogenesis opened new opportunities in the regenerative medicine, however, some limitations regarding exosomes isolation and application remain concerned. In addition, most of the experiments were conducted in preclinical and therefore translation of these results from bench to bed requires more effort in this field. Exosomes from stem cells are a promising tool for the treatment of ischemic diseases. In addition, translation of pre-clinic results into clinic needs further studies in this field.

## Background

Ischemia is characterized by an inadequate blood flow to a local zone as a result of blockage of the blood vessels supplying oxygen and nutrients in the zone. Ischemic is a state that an organ such as the heart, brain, limbs, etc. not receiving sufficient blood and oxygen [1–3]. Ischemic diseases cause disabilities and mortality in humans worldwide [4]. Therefore, recovering blood supply is a hallmark of the treatment of ischemic diseases [5]. Angiogenesis is a process in which new capillaries are formed from vascular bed, therefore it is a key treatment for ischemic diseases [6]. It is well known that hypoxia and insufficient blood supply are the key regulators of angiogenesis, so the idea of therapeutic angiogenesis comprises using a mediator to stimulate the progress of new blood vessels from pre-existing vessels in an ischemic organ/tissue [7]. Current therapies for ischemic diseases include vasodilator and thrombolytic drugs [8] and surgery [9], however, these therapies often are not sufficient for remodeling vascular bed and inducing angiogenesis [10]. In recent years, scientists have focused on using stem cells to induce angiogenesis in ischemic diseases [11–13]. Stem cells can participate to repair the ischemic area by differentiation into cells and/or releasing paracrine factors. Nonetheless, stem cell therapy faces some limitations such as ethical issues, technical issues, immunogenicity, and tumorigenicity [14, 15]. Increasing evidence suggests that extracellular vesicles (EVs) from stem cells facilitate the beneficial effects of cell-therapy for ischemic diseases [16]. EVs are enclosed phospholipid bilayer vesicles releasing from almost cells with the key roles in intracellular communication and physiological as well as pathological processes [16]. Recently, EVs have attracted much attention as cell-based therapeutic agents due to their ability transfer therapeutic biomolecules and facilitating injury repair [17, 18]. A class of EVs is exosomes that facilitate ischemic diseases recovery and angiogenesis through angiogenesis regulatory factors into damaged tissues [19, 20]. Herein, we described the therapeutic function of exosomes from different stem cells in the treatment of ischemic diseases was described. Additionally, we discussed the opportunity and challenges of exosome-therapy as a new cell-free therapeutic agent.

### Angiogenesis

**Fig. 1:**
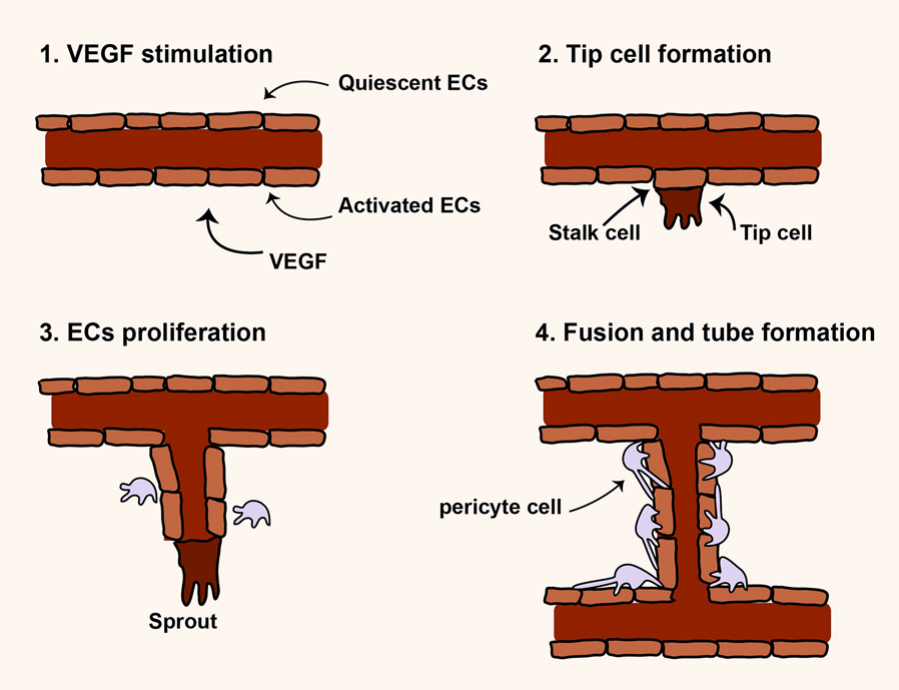
Sprouting angiogenesis. Angiogenesis is a multistep process that different molecules contribute to regulate it [23–25]. At glance, in angiogenesis switch on, MMPs molecules initiate to degrade the extracellular matrix (ECM) and VEGF molecules activate endothelial cells (ECs) to form a distinict and activated ECs, this event often called VEGF stimulation step. In Tip cell formation step, a population of ECs may acquire a distinctive morphology with filopodia that progress into the ECM. Stalk cells are a type of cells behind tip cells support the proliferation and elongation of new tube. In ECs proliferation step, different molecules and signaling molecules increase the proliferation and migration of ECs that cells form sprouting tubules. Tip cells contribute to elongation and guidance of newly formed tube. In fusion and tube formation step, which often called stabilization step, newly formed tubules fuse with each other and such mural cells as pericytes participate in the stabilization of the new capillaries [23–25]

Angiogenesis is a multistep and highly regulated process and essential for growth, development, and repairing damaged tissues [21, 22] (Fig. 1). During angiogenesis, proangiogeneic factors initiate and induce the formation of new vascular network from the blood vessels of a tissue. In regenerative medicine switch on angiogenesis is vital but in some pathological condition such as cancer switch off is essential for cancer inhibition [23–25]. According to literature, angiogenesis may occur by two mechanisms including sprouting and intussusceptions angiogenesis [23, 26]. Hypoxia, a result of ischemia, has been shown to be the main factor for inducing sprouting angiogenesis whereas hemodynamic forces make intussusceptive angiogenesis. Sprouting angiogenesis is well-known as angiogenesis in which two main cell types e.g. endothelial cells (ECs) and mural cells are involved for generation of new vessel [22, 23, 26] (Fig. 1). Whether angiogenesis happens or not depends on the balance between pro and anti-angiogenic factors in the biological environment. High level of proangiogenic factors such as angiopoietins, FGF, VEGF, TGF-α and EGF induce angiogenesis but high level of anti-angiogenic factors including angiostatin, thrombospondin-1/2, interferons, endostatin, and collagen IV inhibit angiogenesis [23–25] As mentioned above, to favor angiogenesis different molecules/signaling pathways are needed to regulate it step by step. The basic steps may involve enzymatic degradation of basement membrane surrounding capillary by matrix metalloproteinases (MMPs), inducing and proliferation of ECs, migration and sprouting of ECs, tubulogenesis, fusion the vessel to each other, trimming of vessel, and pericyte stabilization [23, 27]. Figure 1 illustrates the sprouting angiogenesis.

### Exosomes

**Fig. 2:**
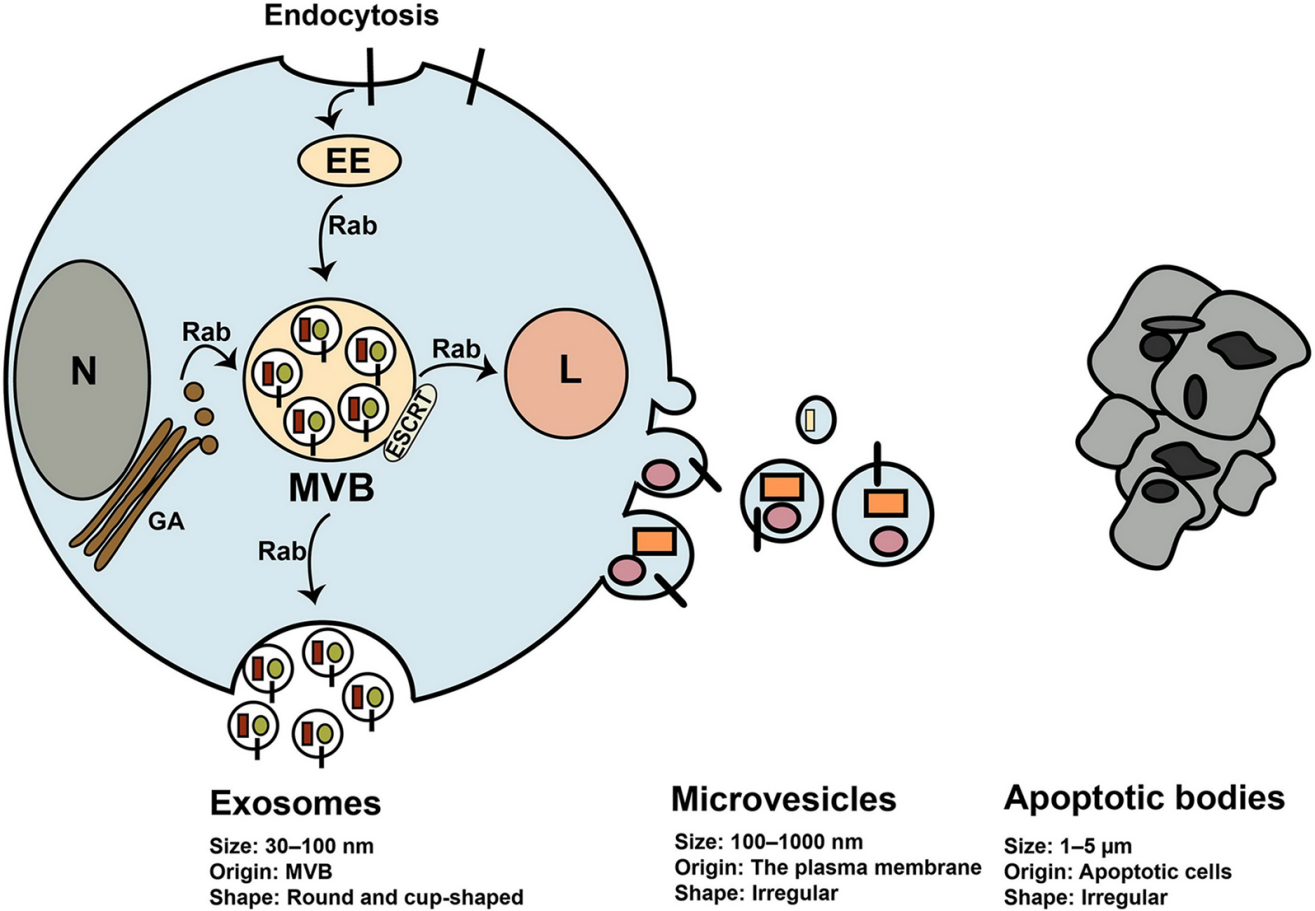
Biogenesis of extracellular vesicles and their characteristics. Exosomes are generated from multivesicle bodies (MVBs) through ESCRT complexes and other molecules. Once generated, MVBs may fuse with the plasma membrane and secrete exosomes out of cell or fuse with the lysosomes (L) for degradation of exosomes. Rab-GTPase proteins regulate intracellular trafficking of MVBs. Different molecules from Golgi apparatus (GA), cytoplasm, and the endosomal pathway are sorted into exosomes

Exosomes belong to EVs family and have a size between 30–150 nm in diameter, posing round shape under electron microscopy [28] (Fig. 2). The International Society for Extracellular Vesicles (ISEV) presented the term extracellular vesicles for vesicles isolated from bio-fluids and cell conditioned media. EVs comprise three class of cell-derived vesicles including exosomes, microvesicles or shedding vesicles, and apoptotic bodies that differ in size and origin [29] (Fig. 2). These vesicles contain many types of biological molecules such as proteins, lipids, and different nucleic acids that transfer them between cells [29]. Thus, EVs contribute to regulating the function of cells, consequently have pivotal roles in normal physiology and also pathophysiological conditions [30, 31]. Many cells including tumor cells, normal cells, and stem cells produce exosomes for establishing cell-to-cell communication [32]. Molecular insight into exosomes biogenesis indicates that exosomes originate from a type of late endosomes called multivesicular bodes (MVBs) located inside the cytoplasm [32, 33] (Fig. 2). MVBs are endo-lysomal compartments where exosomes are generated and loaded with biological components that may fuse with lysosome or fuse with the plasma membrane. Secretion of exosomes occurs when MVBs fuse with the plasma membrane and exosomes release into the extracellular matrix [32, 33] (Fig. 2). Different molecules and complexes such as the endosomal sorting complexes required for transport (ESCRT) located on MVBs regulate exosomes biogenesis and loading [32, 33]. Exosomes comprise different types of bio-molecules gathered from the endosomal pathway, cytoplasm, and even from Golgi apparatus and reticulum endoplasmic. However, typical markers are present on exosomes such as TSG101, Alix, intergrins, and tetraspanins like CD63, CD9, and CD81 [32, 33]. Exosomes derived from stem cells hold great promise for cell-free therapies.

### Angiogenic role of exosomes from different stem cells in myocardial ischemia

**Fig. 3:**
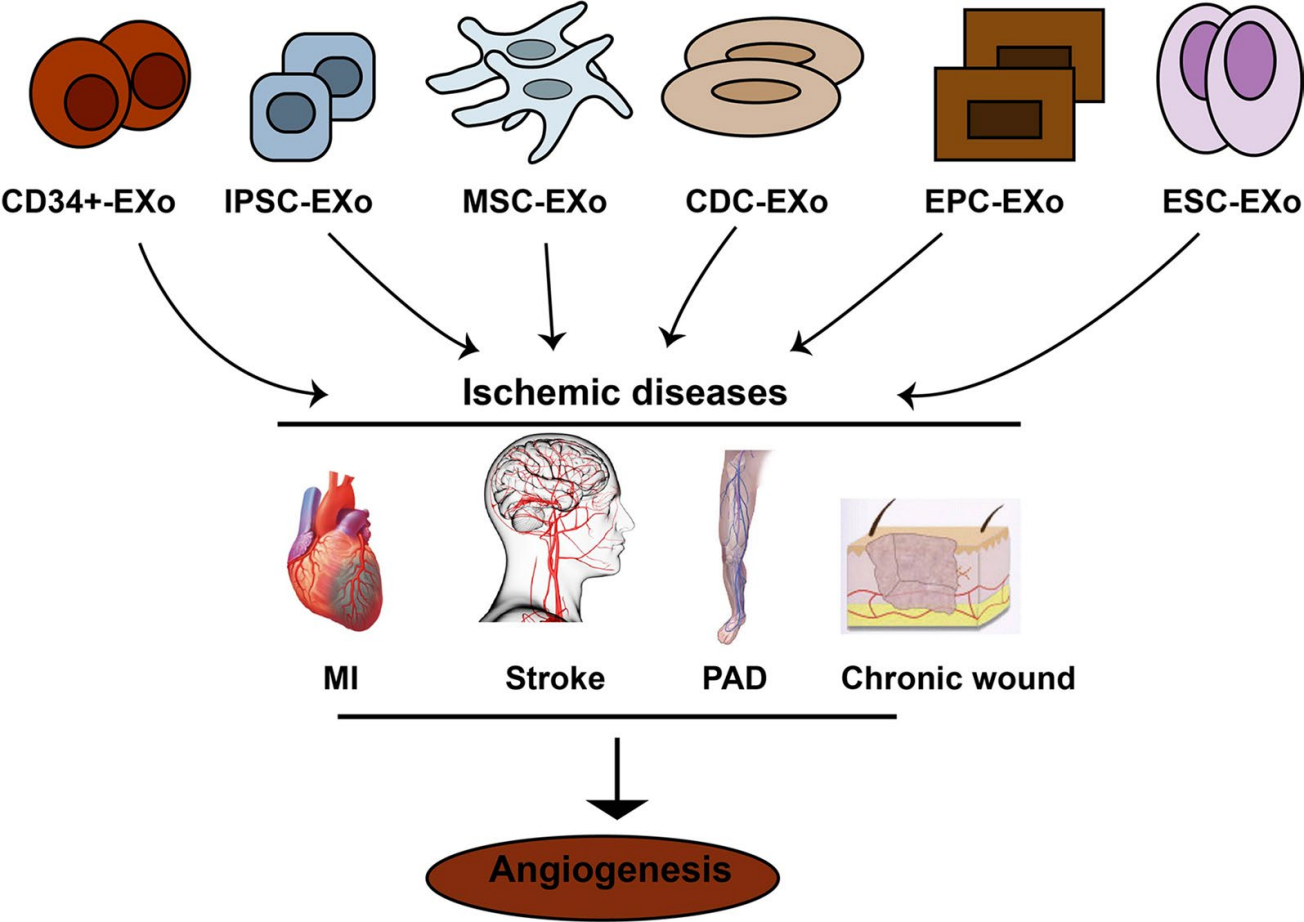
Different stem cells release exosomes improving ischemic diseases by increasing angiogenesis in damaged organ/tissue. MI: myocardial infarction; PAD: peripheral arteries diseases; MSCs-EXo: Mesenchymal stem cells derived exosomes; EPC-EXo: Endothelial progenitor cells derived exosomes; CDC-EXo: Exosomes from cardiospher derived cells. ESC-EXo- Embryonic stem cells derived exosomes; IPSCs-EXo: Induced pluripotent stem cells derived exooxmes

Myocardial ischemia (MI), cardiac ischemia, is a pathological condition that the heart receives oxygen slightly, which consequently leads to a heart attack and serious abnormal heart rhythms [34]. Severe obstruction of the heart's coronary arteries causes MI and consequently reduces blood pump. Treatments for MI include improving blood flow to the heart muscle through angioplasty or bypass surgery [35]. However, this method is suitable for patients with arteries larger than 2 mm in diameter, therefore patients whose arteries are less than 2 mm as well as patients with such indexes as the history of surgeries, deficiency in arteriovenous grafts, diffuse coronary artery diseases remain concerned for revascularization methods [36]. Acute myocardial infarction (AMI) may cause acute coronary block and ischemia–reperfusion injury (IRI), which may lead to acute ischemia and hypoxia in heart muscle cells and cardiomyocytes. Therefore, myocardial remodeling may advance as a result of apoptosis and necrosis in those cells. Scientists believe that stem cell therapy may be an alternative and efficient way to improve MI [37, 38], however, recent advancement in regenerative medicine have revealed that stem cells derivatives may be a more promising tools for the treatment of MI and injured myocardium, promoting myocardial vessel regeneration [39]. Exosomes participate in regeneration and angiogenesis in heart diseases. Exosomes cargo participate in angiogenesis of ischemic diseases (Table 1 and Fig. 3). In this section, the therapeutic role of exosomes from different stem cells source in MI models has been discussed.

**Table 1:** Identified exosomal cargoes of stem cells that improve ischemic diseases

Exosomes from Stem cells	Cargo	Disease
Cardiosphere-derived cells	miRNA-210, miRNA-130a, miRNA-126 miRNA-146a	Myocardial ischemia
CD34^+^ cell	Hedgehog proteins	Myocardial ischemia
Embryonic Stem Cells	Not known	Myocardial ischemia
Induced Pluripotent Stem Cells	Not known	Myocardial ischemia
Mesenchymal Stem Cells	CXCR4, miRNA-21, EMMPRIN	Myocardial ischemia
Stem cells derived from adipose tissue	miRNA-125a	Chronic wound healing
Mesenchymal Stem Cells	miRNA-21-3p, miRNA-126, VEGF	Chronic wound healing
CD34 +	miRNA-126-3p	Chronic wound healing
Stem cells derived from adipose tissue	IL-6	Chronic wound healing
Stem cells derived from adipose tissue	miRNA-181b-5p	Stroke
Mesenchymal Stem Cells	Not known	Stroke
Mesenchymal Stem Cells	VEGF protein, miRNA-210-3p,	Peripheral arterial disease
CD34 + cells	miRNA-126-3p	Peripheral arterial disease

### Exosomes from CDCs

One of cardiac cells is cardiosphere-derived cells (CDCs), which considered as cardiac progenitor cells with the ability to produce different cardiac cells including: endothelial cells, cardiomyocytes, and also smooth muscle cells [40]. A growing body of evidence showed that CDCs can induce angiogenesis and improve indirectly function of the heart in the infarcted myocardium models [41, 42]. Exosomes derived from CDCs (CDCs-EXo) have been found to exert the same result as CDCs do in the treatment of myocardial ischemia [43]. It seems that CDCs obtained from heart tissue have lower immunogenicity compared with other stem cells. Besides, allogeneic CDC-EXo cause low immunogenicity after frequent dosing [44]. Gallet et al*.* reported that CDCs-EXo repaired the necrotic myocardium and induced angiogenesis in a pig model of AMI through intra-tissue injection [45]. CDCs-EXo contain miRNA-146a that reduce apoptosis and increased proliferation of cardiomyocytes in vitro. These exosomes when injected into the heart of the mouse model increased regeneration and angiogenesis [19]. An in vitro experiment showed that hypoxic CDCs release exosomes with distinct miRNAs. Namazi et al*.* found that these exosomes contain a high level of miRNA-210, miRNA-130a, and miRNA-126 that induce tube formation and promote angiogenesis in human umbilical vein endothelial cells (HUVECs) [46]. Exosomes from human CDCs induced angiogenesis in HUVECs and decreased the apoptosis rate of cardiomyocytes [47].

### Exosomes from ESCs

Embryonic stem cells (ESCs) can release exosomes affecting the function of target cells. Khan et al*.* isolated exosomes from ESCs and injected them intramyocardially into mice model of AMI. An Immunohistochemical experiment showed that the vessel density was significantly increased in transplanted myocardium [48]. Exosomes from human ESC-derived cardiovascular progenitors showed the cardioprotective effects in mice model of the heart failure. Further analysis revealed that 927 genes were up-regulated in the exosomes-treated hearts, of which the majority associated with cardiac function [49]. Similarly, exosomes isolated from human ESC-derived mesenchymal Stem Cells (MSCs) caused a decrease in infarct size in MI/reperfusion (MI/R) injury in the mouse model. The molecular study indicated that the levels of ATP and NADH, phosphorylated (p)-Akt, and p-GSK-3β increased, while oxidative stress and p–c-JNK decreased in MI/R hearts [50].

### Exosomes from CD34^+^ cells

Hematopoietic stem/progenitor cells usually express CD34 marker and this marker progressively declines or even disappear with differentiation into matured cells. Intramyocardial injection of autologous CD34 + cells improved myocardial perfusion in patients associated with AMI by promoting angiogenesis [51]. Similarly, exosomes from CD34 + cells (CD34 + -EXo) supported ECs viability and proliferation and also promoted Matrigel-based tube formation [106]. Mackie et al*.* showed that CD34 + -EXo contain a high level of sonic hedgehog (Shh) molecules that improved function of the heart in AMI hearts [52].

### Exosomes from MSCs

MSCs are beneficial cells as they can participate in the regeneration of damaged cells/tissues. Exosomes from MSCs (MSCs-EXo) can be uptake by ECs, which in turn induces angiogenesis. Teng et al*.* found that injection of MSCs-EXo can increase angiogenesis and increase blood vessel density in an animal infarcted myocardium model [53]. Exosomes from CXCR4 overexpressing MSCs activate Akt signaling in cardiomyocyte and inhibit cardiomyocyte apoptosis as well as increase VEGF expression and vessel formation in vitro [54]. In a rat MI model, these exosomes supported cardiac functional renewal by inducing angiogenesis, decreasing infarct size, and increasing cardiac restoration [54]. Vrijsen et al. conducted in vitro and in vivo experiments by exosomes from bone marrow-derived MSCs and reported that these exosomes promoted the migration of ECs and tube formation via ERK/Akt signaling. In keeping, they mixed exosomes with Matrigel plug and then implanted into mice subcutaneously. Results showed that the migration of ECs and tube formation increased in exosomes-mixed Matrigel plug. Further analysis showed that these exosomes enriched with extracellular matrix metalloproteinase inducer (EMMPRIN) proteins, which are responsible for the angiogenic effect of exosomes [55]. Exosomes from umbilical cord MSCs have shown to increase migration of ECs and tube formation that correlated with up-regulation of Bcl-2 family in ECs [56]. Wang and co-workers indicated that exosomes from endometrium derived MSCs more effectively increased microvessel density in a rat model of AMI as compared to exosomes from bone marrow and adipose-derived MSCs. These exosomes contain abundantly miRNA-21 that exert cardioprotection through the tensin homolog (PTEN)/ Akt pathway [57].

### Exosomes from iPSCs

Recently, scientists have focused on the application of induced pluripotent stem cells (iPSCs) in tissue regeneration. IPSCs are produced by reprogramming somatic cells into the embryonic-like pluripotent state with differentiation characteristics. Both iPSCs and their exosomes iPSC-EXo show promising results in heart failure treatment by inducing ECs migration and tube formation. For example, when iPSV-Exo were injected into the hearts of mice, they increased regeneration of capillaries in the infarct zone and border zone of the heart failure and repaired heart tissues in a mouse model [58]. Exosomes from iPSCs derived cardiovascular progenitor cells (iPSC-CPC) increased the migration and tubulogenesis in HUVECs in vitro. Furthermore, these exosomes considerably restored the function of chronic heart failure by improving left ventricular ejection fraction and reducing left ventricular volumes [59].

### Angiogenic role of exosomes from different stem cells in chronic wound healing

Chronic wounds are wounds that has failed to progress healing in 30 days with complex pathogenesis [60]. The main causal factors may comprise pressure [61], trauma [62], diabetic ulcers [63], arteriosclerosis [64], lower extremity wounds and venous ulcers [65], ischemia [66]. Ischemia plays a key role in the formation and persistence of the wound, particularly when it happens frequently or when associated with senescence [67]. In the following, ischemia leads to inflamed tissue and induces cells to produce factors that fascinate inflammatory immune cells [67]. These wounds result in emotional stress and physical effects on patients and also represent a significant burden to patients and even to the healthcare system [68]. Complications observed in chronic wound healing related to lack or insufficient angiogenesis [69]. Additionally, insufficient and inappropriate angiogenesis associated with acute wounds may progress to chronic wounds [70]. Treatment of the various chronic wound differs somewhat, suitable treatment ways should address the problems decrease ischemia, bacterial load, irrigation, debridement and increasing oxygenation, warming, moisture, cellular proliferation, and healing factors [71–73]. Exosomes from stem cells have been demonstrated to improve chronic wounds through increasing angiogenesis (Fig. 3). In this section, the pivotal role of exosomes from a different stem source was described.

### Exosomes from EPCs

Endothelial progenitor cells (EPCs), which are present in the umbilical cord blood, bone marrow, and peripheral blood can participate in facilitating angiogenesis [74, 75]. In vivo experiments demonstrated that transplantation of EPC-EXo could accelerate skin wound healing in diabetic rats by positively modulating vascular ECs function [76]. There is an evidence that exosomes from EPCs (EPC-EXo) increase angiogenesis and improve the healing of diabetic skin wounds in an animal model [77]. Besides, in vitro study indicated that EPC-EXo augmented the migration and proliferation rate of vascular ECs and enhanced expression of angiogenic factors like VEGF and HIF-1α [77]. Further in vitro scrutiny showed that these exosomes up-regulated the expression of ANG-1, E-selectin, eNOS, IL-8, VEGFA, VEGFR-2, HIF- 1a, PDGFA and CXCL16, but inhibited expression of PDGFB and MMP-9, which finally increased angiogenesis in HMECs [78]. The same results have been reported in another study where authors declared that these exosomes promoted angiogenesis both in vitro and in vivo diabetic model. They showed that expression of ANG-1, aFGF, IL-8, E-selectin, eNOS, VEGFR-2, VEGFA, and CXCL-16 increased, however the expression of MMP-9 decreased in HMECs [79]. However, exosomes cargoes could deliver into ECs, which regulated genes, remains yet unclear.

### Exosomes from ADSCs

One of the promising stem cells in regenerative medicine is stem cells derived from adipose tissue (ADSCs). These cells are self-renewal and have multidirectional differentiation potential [80]. Recent evidence suggests that ADSCs can considerably increase capillary density in chronic wounds [81, 82]. Exosomes from ADSCs (ADSC-EXo) represent the same effect as ADSCs exert on the regeneration process. For example, Liang et al*.* declared that ADSC-EXo can induce angiogenesis both in vitro and in vivo. They showed that these exosomes deliver miRNA-125a to ECs, which regulate proangiogenic genes such as Flk1 and Ang1 as well as anti-angiogenic genes TSP1, Vash1, and DLL4, consequently, improving wound healing [83]. In addition, exosomes from hypoxia-treated ADSCs can increase tubulogenesis and angiogenesis in ECs through the PKA signaling pathway. These exosomes may up-regulate pro-angiogenic genes like Flk1, Angpt1, and VEGF, while down-regulate Vash1 as anti-angiogenic gene [83]. These results could be useful for treatments of Ischemic diseases.

### Exosomes from MSCs

A growing body of studies showed that the paracrine factors derived from MSCs can induce angiogenesis in the wound through activating ECs [84, 85]. Exosomes are a vital paracrine factor of MSCs that are promising MSCs-based therapies. For example, Shabbir et al*.* showed that MSCs-EXo can be uptake by HUVECs, subsequently promoting angiogenesis in vitro [86]. Further studies declared that MSC-EXo induce angiogenesis in ECs by regulating different signaling pathways such as STAT3, AKT, and ERK. In downstream, these pathways may up-regulate the expression of bFGF, VEGF, and TGF-β, which therefore improve endothelial angiogenesis [86]. Hu et al*.* reported that exosomes from MSCs of umbilical cord increased angiogenesis in human mammary epithelial cells (HMECs) in vitro and in mice model of skin wounds [87]. In keeping, they found that exosomal miRNA-21-3p regulates PI3K/Akt and ERK1/2 signaling inside ECs, therefore increase angiogenesis. Similar results have been reported by Zhang et al*.* who declared that exosomes from MSCs of umbilical cord induce angiogenesis in EA.hy926 cells in vitro, in addition, they showed that these exosomes transport Wnt4 to target cells and increase wound healing in burn wounds model [88]. Wnt4 can activate β-catenin in ECs and exerts angiogenic effects. MSCs-EXo could deliver Wnt3a into dermal fibroblast and increase proliferation, migration, and angiogenesis in vitro [89]. Exosomes derived from placenta MSCs can promote angiogenesis in HMECs in vitro and also in vivo in an ischemic injury model [90].

### Exosomes from iPSCs

iPSCs-EXo have been shown to significantly increased vessels of full-thickness excisional skin wounds in diabetes mice [91]. Furthermore, iPSCs-EXo can promote proliferation, migration, and tubulogenesis of HUVECs and fibroblasts in dose-dependently in vitro. Also, expression and secretion of type I, III collagen were increased in fibroblast. These exosomes increased regeneration of vessels in a rat skin full-thickness defect model [79]. These results show iPSCs-EXo can facilitate cutaneous wound healing however, these data are insufficient and underlying mechanisms have not been noticeably elucidated.

### Angiogenic role of exosomes from different stem cells in PAD

Peripheral artery disease (PAD) is a pathological condition on the circulatory system in which tightened arteries decrease blood flow to body limbs [92]. In advance peripheral artery disease legs or arms frequently legs face reduced blood supply to keep up with demand. This may cause claudication symptoms and affects life quality and burden of socioeconomic problems [93]. PAD is also likely to be an indication of an accumulation of fatty pledges in arteries so-called atherosclerosis. In response, atherosclerosis may slender arteries and lessen blood supply to legs and, sometimes, to arms [92]. Recent progress in the field of stem cells has led to a renewed interest in the application of their exosomes in inducing angiogenesis in PAD. For instance, it was demonstrated that exosomes from iPSC-derived MSCs (iMSCs-EXo) endorsed angiogenesis after injection into ischemic limbs of mice model [94] (Table. 1; Fig. 3). EVs isolated from bone marrow MSCs can boost the blood vessels form in the ischemic limb in vivo. These EVs are enriched with VEGF protein and miRNA-210-3p that up-regulated expression of angiogenic genes such as VEGFR1 and VEGFR2 in ECs [95, 96]. Du et al*.* boosted the angiogenic potential of exosomes with a nitric oxide-releasing polymer. They co-cultured MSCs with this polymer and harvested exosomes. In keeping, these exosomes enhanced angiogenesis in HUVECs in vitro and in hind limb ischemia in a Murine model [97]. Molecular analysis showed that a high level of VEGF and miRNA-126 cargo in iMSCs-EXo was responsible for promoted angiogenesis [97]. Furthermore, CD34 + cells release exosomes transferring miRNA-126-3p into target cells that repair ischemic hind limb [98]. MiR-126-3p can target SPRED1 expression and participate in up-regulation of genes involved in angiogenic pathways, which in turn promote angiogenesis [98].

### Angiogenic role of exosomes from different stem cellsin stroke

A stroke happens when the bloodstream to part of the brain is broken up or reduced, depriving brain cells of receiving oxygen and nutrients and then brain cells begin to die rapidly [99]. Stroke is characterized by cerebral ischemic and hemorrhagic damage and most strokes (87%) are ischemic strokes [100]. Ischemia induces degeneration and necrosis in neural tissue, consequently induces a permanent loss in the ischemic core area [101]. The therapeutic methods for stork comprise use of intravascular thrombectomy and tissue plasminogen activator thrombolysis. However, these methods are time-limited and should be done only a few hours [102]. Furthermore, neurological dysfunction may remain concerned after getting operational thrombolytic therapy. In this regard, exosomes as regenerative agents have emerged as alternative/ supplementary therapy in preclinical experiments [103]. MSCs-EXo play an important role in the improvement of adverse effect of stork [19, 104] (Fig. 3). For example, Xin et al*.* designed a middle cerebral artery occlusion of rats as stork model and found that bone marrow MSCs-EXo promoted ECs proliferation and significantly increased the capillary network [104]. Yang et al*.* examined the therapeutic function of ADSCs-EXo in vitro and in vivo stork models. They found that these exosomes transfer miRNA-181b-5p to brain microvascular endothelial cells (BMECs) cultured in oxygen–glucose deprivation condition [105] and increased tubulogenesis. Further analysis confirmed that this miRNA up-regulated HIF-1α and VEGF but down-regulated expression of transient receptor potential melastatin 7 (TRPM7) in BMECs. A clinical trial with Identifier number NCT03384433 has been recorded on ClinicalTrials.gov database (https://www.clinicaltrials.gov), which aimed to investigate the therapeutic effect of allogenic MSCs-EXo in patients with acute ischemic stroke. These studies suggest that exosomes may provide a new therapeutic attitude for stroke therapy.

### Opportunity and limitations

A considerable amount of literature has been published on exosomes. These studies showed that exosomes are a favorable opportunity for the treatment of ischemic diseases as they promote angiogenesis. Especially, exosomes from stem cells can increase proliferation, migration, and angiogenesis of cells involved in angiogenesis. The application of exosomes in ischemic diseases may have many advantages. For instance, exosomes from stem cells contain pro-angiogenic factors or/and angiogenesis-related regulators such as signaling molecules and miRNAs that can be directly delivered into cells, promoting angiogenesis [95, 106]. Besides, the scientist can manipulate exosomes cargo as a platform for delivering biomolecules to target cells through different ways such as engineering or preconditioning parental cells [107]. These exosomes are called artificial or optional exosomes with the targeted application for different diseases [107]. Another advantage is the biological nature of exosomes that make them a natural nanocarrier for the biological components and also drugs [108]. As they harbor lipid bilayer encompassing biological material; not only they preserve cargoes from enzymatic degradation but also passively and specifically deliver them into target cells. In addition, exosomes can be useful for personalized medicine [109, 110]. In this regard, exosomes can be produced from autologous stem cells even from iPSCs that undergone gene-editing or cargo-loading manipulating process. This process holds great promise for the treatment of ischemic diseases. Moreover, it seems that exosomes especially from MSCs are safe and non-tumorigenic, even allogeneic source is not immunogenic for the host [111]. Despite this opportunity, some limitations remain concerned regarding exosomes isolation, definition, and also application [112]. Although extensive research has been carried out on exosomes, however, researchers use different ways to isolate exosomes. Seemingly, exosomes isolation methods can affect the results and outcome of experiments. Another problem is the definition of exosomes. As mentioned previously, ISEV released guidelines in 2014 and 2018 about EVs describing exosomes/EVs isolation, characterization, and definition [112, 113]. Some studies may not use ISEV updated guidelines for exosomes. EVs may overlap on size and even in markers, which may affect results, so researchers may unintentionally use the term exosomes. Select a safe and confident source of exosomes is another concern that researchers should attention to providing exosomes associated with angiogenic property and at the same time are non-immunogenic and non-tumorigenic. In addition, exosomes administration ways are vital for getting promising results. Different approaches have been examined by researchers to deliver exosomes into target tissues [15]. Therefore, exosomes transplanted into the target tissues may represent short-term effects because of having a short half-life and quick clearance by the immune cells [114]. Hydrogel-based exosomes delivery may be a promising way to load exosomes into damaged tissue for inducing angiogenesis [115]. Consequently, it is needed to explore the efficacy and sensitivity of administration ways for appropriate translation of results into the clinic [116]. Another concern may be related to the uptake capacity of target cells. In this regard, it seems that an increase in cellular uptake of exosomes by target cells may increase angiogenesis rate [117]. Thus, this limitation remains to be considered in further studies. Further data collection is required to determine exactly how we improve our knowledge of exosomes biology and application for translation into the clinic.

## Conclusion

The evidence from this study suggests that exosomes from stem cells have angiogenic potential that can improve ischemic diseases like MI, chronic wound, and PAD. Despite promising results, most of these studies performed in pre-clinical and results from clinical trial remain to be elucidated in forthcoming studies. Exosome-based therapy represents advantages, however, some limitations are associated with exosome-related methods and exosomes-bio-application. Further experimental investigations are needed to solve these limitations, therefore outcomes move from bench to bed.

## Data Availability

The datasets used and/or analyzed during the current study are available from the corresponding author on reasonable request.

## Abbreviations

EVs: Extracellular vesicle
MSCs-Exo: Mesenchymal Stem Cells derived Exosomes
ECs: Endothelial cells
MMPs: Matrix metalloproteinases
ISEV: International Society for Extracellular Vesicles
MVBs: Multivesicular bodes
MI: Myocardial ischemia
AMI: Acute myocardial infarction
IRI: Ischemia-reperfusion injury
CDCs: Cardiosphere-derived cells
CDCs-EXo: Exosomes derived from CDCs
HUVECs: Human umbilical vein endothelial cells
ESCs: Embryonic stem cells
CD34+-EXo: Exosomes from CD34+ cells
Shh: Sonic hedgehog
MSCs-EXo: Exosomes from MSCs
iPSCs: Induced pluripotent stem cells
EPCs: Endothelial progenitor cells
EPC-EXo: Exosomes from EPCs
ADSCs: Stem cells derived from adipose tissue
DSC-EXo: Exosomes from ADSCs
HMECs: Human mammary epithelial cells
BMECs: Brain microvascular endothelial cells
TRPM7: Transient receptor potential melastatin 7
PAD: Advance peripheral artery disease
